# AKT Hyperphosphorylation and T Cell Exhaustion in Down Syndrome

**DOI:** 10.3389/fimmu.2022.724436

**Published:** 2022-02-10

**Authors:** Daphne Peeters, Ingrid Pico-Knijnenburg, Douwe Wieringa, Mandana Rad, Roos Cuperus, Madelon Ruige, Frank Froeling, Gerda W. Zijp, Mirjam van der Burg, Gertjan J. A. Driessen

**Affiliations:** ^1^ Department of Pediatrics, Juliana Children’s Hospital, The Hague, Netherlands; ^2^ Department of Pediatrics, Laboratory for Pediatric Immunology, Willem-Alexander Children’s Hospital, Leiden University Medical Centre, Leiden, Netherlands; ^3^ Department of Pediatric Anaesthesiology, Juliana Children’s Hospital/Haga Teaching Hospital, The Hague, Netherlands; ^4^ Department of Pediatric Urology, Juliana Children’s Hospital, The Hague, Netherlands; ^5^ Department of Paediatric Surgery, Juliana Children’s Hospital, The Hague, Netherlands; ^6^ Department of Paediatrics, Maastricht University Medical Centre, Maastricht, Netherlands

**Keywords:** down syndrome, AKT pathway, T cell exhaustion, immunophenotyping, immunodeficiencies, activated PI3 kinase delta syndrome

## Abstract

Down syndrome (DS) is associated with increased susceptibility to infections, auto-immunity, immunodeficiency and haematological malignancies. The exact underlying immunological pathophysiology is still unclear. The immunophenotype and clinical characteristics of DS resemble those of Activated PI3K Delta Syndrome (APDS), in which the PI3K/AKT/mTOR pathway is overactivated. We hypothesized that T cell exhaustion and the hyperactivation of the AKT signalling pathway is also present in immune cells of children with DS. In this observational non-interventional cohort study we collected blood samples of children with DS (n=22) and healthy age-matched controls (n=21) for flowcytometric immunophenotyping, phospho-flow AKT analysis and exhaustion analysis of T cells. The median age was 5 years (range 1-12y). Total T and NK cells were similar for both groups, but absolute values and transitional B cells, naive memory B cells and naive CD4+ and CD8+ T cells were lower in DS. pAKT and AKT were increased for CD3+ and CD4+ T cells and CD20+ B cells in children with DS. Total AKT was also increased in CD8+ T cells. Children with DS showed increased expression of inhibitory markers Programmed cell dealth-1 (PD-1), CD244 and CD160 on CD8+ T cells and increased PD-1 and CD244+ expression on CD4+ T cells, suggesting T cell exhaustion. Children with DS show increased pAKT and AKT and increased T cell exhaustion, which might contribute to their increased susceptibility to infections, auto immunity and haematological malignancies.

## Introduction

Trisomy 21 is the most common chromosomal abnormality and is associated with a variety of clinical conditions, including cardiac pathology, auto-immunity, immunodeficiency, haematological malignancy and neurological abnormalities such as early dementia (1–4). Individuals with Down syndrome (DS) are more susceptible to infections, especially those of the respiratory tract (5). The increased risk of infections can partly be explained by abnormal anatomy of the ENT region and respiratory tract, but defects in the adaptive immune response likely contribute, such as a decrease in naive and memory B and T cells, decreased primary antibody responses as well as a skewing of the different serum immunoglobulins with an increase of IgG and decrease of IgM (6).

The underlying pathophysiology of the immunological problems is still unclear. Schoch et al. (7) showed an increased expression of the inhibitory marker Programmed cell dealth-1 (PD-1) on CD4+ T cells, CD8+ T cells and regulatory T cells, suggesting an increased state of functional anergy in Down syndrome. The combination immunodeficiency, auto-immunity, the propensity to develop haematological malignancies and T cell anergy/exhaustion has previously been described in the primary immunodeficiency activated PI3K Delta Syndrome (APDS) (8–11). In this primary immunodeficiency the PI3K/AKT/mTOR pathway is overly activated. Increased intracellular AKT-activity stimulates metabolism, cell proliferation, survival and growth and at the same time deregulates the humoral immune response and induces T cell anergy. Interestingly, hyperactivation of this pathway has been reported in the frontal cortex of individuals with DS who suffer from early dementia when compared to controls (12). However, increased PI3K/AKT activity has not been explored in immune cells of patients with DS. We hypothesized that the AKT/mTOR pathway is also hyperactivated in lymphocytes of patients with DS, which might offer an explanation for the observed T cell exhaustion, immunodeficiency and immune dysregulation. Our aim was therefore to explore the extent of T cell exhaustion and hyperphosphorylation of the PI3K/AKT pathway in children with DS compared to healthy age-matched individuals.

## Methods

### Materials and Method

In this observational non-interventional cohort study with a cross-sectional design, the immunological characteristics of children with DS were compared with those of healthy age-matched controls. Blood samples were collected during regular healthcare check-ups in the Juliana Children’s Hospital from May 2018 to January 2020. The acquired blood sample volume was adjusted to the weight of the child. Clinical data on age, gender, premedical history, and use of medicines were collected from the electronic health record. This study was conducted in accordance with the Declaration of Helsinki. Prior to commencing this study, approval was obtained from the Medical Ethics Committee Zuidwest Holland.

### Study Population

Children with DS (0-17 years) were asked to participate whenever in the outpatient department blood samples were taken for regular healthcare reasons. Blood samples were only taken if children with DS did not experience an episode of fever or showed other signs or symptoms of an infection at that moment. Healthy age-matched controls were recruited when undergoing a minor surgical procedure. Healthy children were matched with a maximum difference of one year with DS patients. Children were excluded in case of an active infectious disease, malignancy, mosaic DS, or auto-immune disease other than those associated with DS (such as coeliac and thyroid disease). Children who suffered from recurrent respiratory tract infections or getting surgery because of this reason, were excluded as a healthy control.

Before enrolment, parents or other legal caregivers and children ≥12 years of age were asked for informed consent.

### Blood Samples

**Figure 1:**
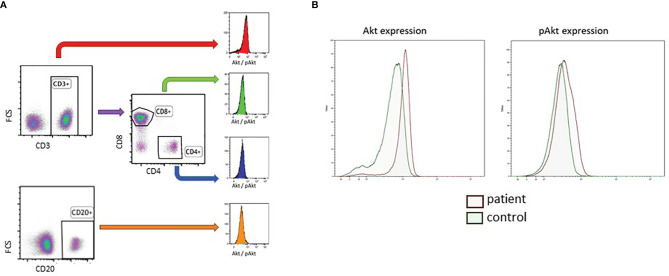
Gating strategy of B cells and T cell subset for the determination of baseline AKT and pAKT expression in DS and healthy controls. **(A)** Gating strategy of CD3+CD4+ T cells, CD3+CD8+ T cells and CD20+ B-cells. **(B)** Representative phospho-flow plots for AKT and pAKT in unstimulated CD3+ T cells in a DS patient and age matched healthy control.

White blood cell (WBC) count was measured on the Sysmex XP-300 in fresh blood. Before being frozen, peripheral blood mononuclear cells (PBMCs) were separated using Ficoll-paque within 24 hours after collection of the blood sample. Procedures were similar to those previously described (13). Flowcytometric immunophenotyping was performed on fresh blood using BD FACSCanto-II flowcytometer. Analysis was done with BD FACSDiva software. An example of the gating strategy for T cell subsets and B cells is shown in **Figure 1A**. Antibody levels and immunization responses were not available for our cohort of DS patients. PBMCs were used for phospho-flow analysis. After washing and staining, cells were measured on the BD LSR-II flowcytometer. Analysis was done with Kaluza Analysis software and statistics were performed using Graphpad Prism software. For the level of exhaustion, frozen PBMC samples were washed, stained and analysed using BD FACSDiva software. To examine the extent of T cell exhaustion, the expression of inhibitory markers PD-1, CD244 and CD160 and their co-expression were examined on CD4+ and CD8+ T cells. The marker CD57 was used to identify senescent T cells or terminally differentiated T cells with reduced proliferative capacity (14, 15). See **Supplementary File** for more detailed information on flowcytometric immunophenotyping, and exhaustion assays.

### Phospho-Flow

PBMCs were thawed in cold FCS and RPMI, washed and resuspended in PBS with BSA and azide. Cells were counted with Sysmex XP-300. Cells were washed in pure and cold PBS. FVD (Viability staining Protocol) (InvitrogenTM) coloring (1000x dilution) was performed to irreversibly label dead cells prior to fixation and permeabilization, to exclude dead cells when targeting intracellular components. Cells were incubated for 30min on coolblock (4°C) and shaking platform before being washed. Cells were divided in two wells per patient/control. Cells were stained with CD8 BV510 (BD Biosciences) and CD20 BV421 (Biolegend, San Diego, CA, USA) and incubated for 15min in a 37°C stove. Cells were fixated with BD Cytofix Fixation Buffer and incubated for 10min at 4°C. Unstimulated cells were washed and permeabilized with BD Phosflow Perm Buffer III and incubated for 30min at -20°C in the dark. Cells were washed and stained with AKT AF488 or pAKT AF488 (Both BD Biosciences), CD3 APC (Beckman Coulter, Brea, CA, USA), CD4 PE and CD69 PE-Cy7(BD Biosciences) and CD25 PerCP-Cy5.5 (Biolegend, San Diego, CA, USA) and incubated for 15 min at RT in the dark. Cells were washed and AKT or pAKT was measured on the BD LSR-II flowcytometer. Analysis was done with Kaluza Analysis software and statistics were performed using Graphpad Prism software. A representative example of a phospho-flow plot is shown in **Figure 1B**. FMO (fluorescence minus one) controls were used to check for background staining (**Supplementary Figure 2**).

### Statistical Analysis

Descriptive analysis of clinical data was performed using SPSS version 24. For Flowcytometric immunophenotyping, exhaustion phenotyping, pAKT and AKT were compared using two-tailed t-tests or Mann-Whitney U test depending on whether the data was Gaussian distributed. Statistical analyses were performed using GraphPad Prism software (GraphPad Prism Software, San Diego, CA, USA). *P*-values smaller than 0.05 were considered statistically significant. The total number of subjects was too small to perform a meaningful subgroup analysis for different age groups.

## Results

### Study Population

In total, 22 children with DS and 21 healthy age-matched controls were enrolled. An overview baseline characteristics of patients and healthy controls, including co-morbidity, is shown in **Supplementary Table 1**. The median age was 5 years (range 1-12 years). As expected, (previous) cardiac pathology was the most common comorbidity in children with DS (64%). Due to selective enrolment, healthy controls had minor comorbidities and none of them suffered from frequent infections or auto-immune disease. Children with DS suffered more frequently from respiratory infections than their healthy peers, as illustrated by previous ENT surgery in 64% and hospitalization for a lower respiratory tract infection in 36%. None of the children suffered from bronchiectasis. Auto-immune disease was present in 14%. Current or past haematological malignancies were not present in our cohort. All immunological analyses were performed for every participant except for one child with DS and one healthy control, in whom it was not possible to collect sufficient blood to perform all analyses.

### Lymphocyte Subsets

Circulating levels of total T cells (CD3+ cells) and NK cells (CD16.56+ cells) were similar for children with DS and controls. However, B cell (CD19+) levels were reduced in children with DS (*p*<0.005). 41% of children with Down syndrome had absolute B cells counts below the 5^th^ percentile of the range for the corresponding age (16). True counts of lymphocyte subsets are shown in **Supplementary Table 2**. Flowcytometric immunophenotyping was done in 22 DS patients and 20 healthy controls.

### B Cell Differentiation

**Figure 2:**
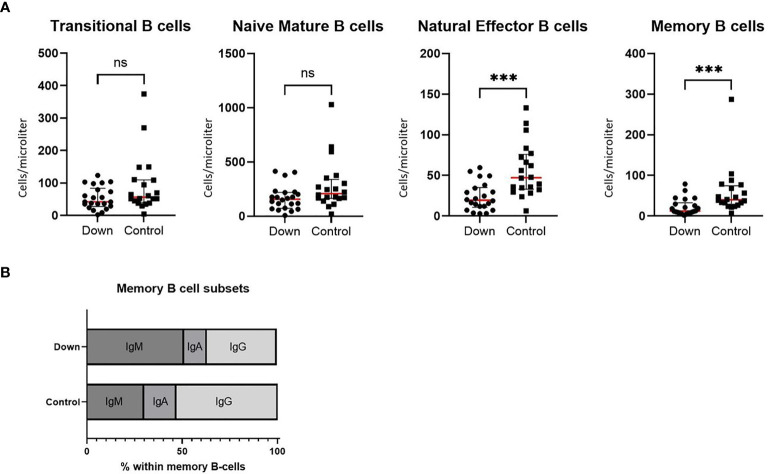
Immunophenotyping of B cells in 22 children with Down syndrome and 20 healthy age-matched controls. **(A)** Absolute values of transitional B cells (CD38high/CD24high) and naive mature B cells (CD38dim/CD24dim/IgD+/CD27-) did not differ between children with Down syndrome and healthy controls, whereas natural effector B cells (CD38dim/IgD+/CD27+) and memory B cells (CD28dim/IgD-/CD27+) were significantly decreased in children with Down syndrome (resp. *p*=0.0002 and *p*=0.0004). Median and interquartile ranges are indicated (red lines represent the median). **(B)** In the subsets of memory B cells, children with Down syndrome have increased IgM memory B cells (*p*<0.0001) and decreased IgA (*p*=0.0003) and IgG (*p*<0.0001) memory B cells. ***p < 0.001; *ns p* > 0.05.

Whereas absolute values of transitional B cells and naive mature B cells do not differ between children with Down and healthy controls, natural effector cells and memory B cells are significantly lower (**Figure 2A**). Children with Down syndrome also showed less switching within memory B cells, resulting in relatively more IgM memory B cells (**Figure 2B**). We did not find a significant difference in the percentage of CD38-CD21- cells between children with DS and healthy controls (data not shown).

### T Cell Differentiation

**Figure 3:**
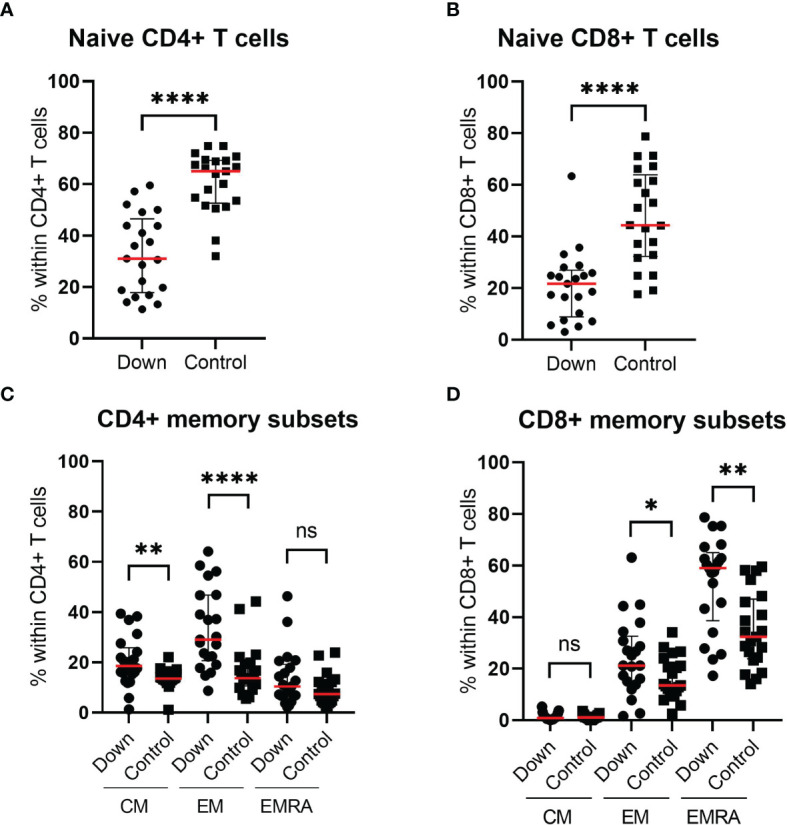
Immunophenotyping of the T cell compartment of 21 children with Down syndrome and 21 healthy age-matched controls. Children with Down syndrome have significantly lower naive CD4+ T cells (CD4+CD45RA+CCR7+) **(A)** and lower naive CD8+ T cells (CD8+CD45RA+CCR7+) **(B)** compared to healthy controls. **(C)** Within the CD4+ T cells, the proportion of CM and EM cells is increased in children with Down syndrome. **(D)** For CD8+ T cell distribution, there is no significant difference in the percentage CM cells, however, children with Down syndrome have increased EM and EMRA. Median and interquartile ranges are indicated. *p <0.05; **p < 0.01; ****p < 0.0001; ns p > 0.05.

Whereas the total CD4+ and CD8+ T cell counts did not differ significantly between both groups, we did find differences in the subset distributions. The percentage of naive CD4+ T cells was 33.0 ± 15.51 (mean ± SEM) in children with Down syndrome compared to 60.93 ± 2.53 in healthy controls (*p*<0.0001; **Figure 3A**). This difference was also apparent for naive CD8+ T cells: 21.1 ± 3.0 in DS versus 47.4 ± 4.0 in controls (*p*<0.0001; **Figure 3B**). Within the CD4+ T cell compartment, children with DS showed elevated levels of CD45RA-CCR7+ central memory T cells (CM) (*p*=0.006) and CD45RA-CCR7- effector memory T cells (EM) (*p*<0.0001), whereas CD45RA+CCR7- effector memory cells re-expressing RA (EMRA) were similar in both groups (**Figure 3C**). Another shift was seen for CD8+ T cells (**Figure 3D**). Children with DS had similar CD8+CM levels, but increased CD8+ EM (*p*=0.35) and CD8+ EMRA cells (*p*=0.0015).

### Increased AKT Phosphorylation

**Figure 4:**
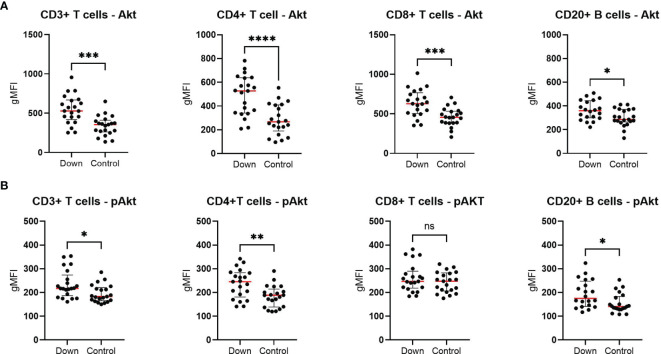
Phospho-flow analysis in 21 children with Down syndrome and 21 healthy age-matched controls. **(A)** The total AKT levels were elevated in all different cells for children with Down syndrome. **(B)** In children with Down syndrome, pAKT levels were increased in CD3+, CD4+ and CD20+ cells, but not in CD8+ T cells. Median and interquartile ranges are indicated. *p < 0.05; **p < 0.01; ***p < 0.001; ****p < 0.0001; ns p > 0.05.

In order to determine the levels of total AKT and activated AKT by phosphorylation (pAKT), we performed a phospho-flow assay in T cell subsets (CD3+, CD4+ and CD8+) and B cells (CD20+). Children with DS (n=21) showed elevated levels of AKT in all cells, especially in the T cell compartment compared to 21 healthy controls (**Figure 4**). Except for CD8+ cells, pAKT levels were increased in all subsets.

### T Cell Exhaustion

**Figure 5:**
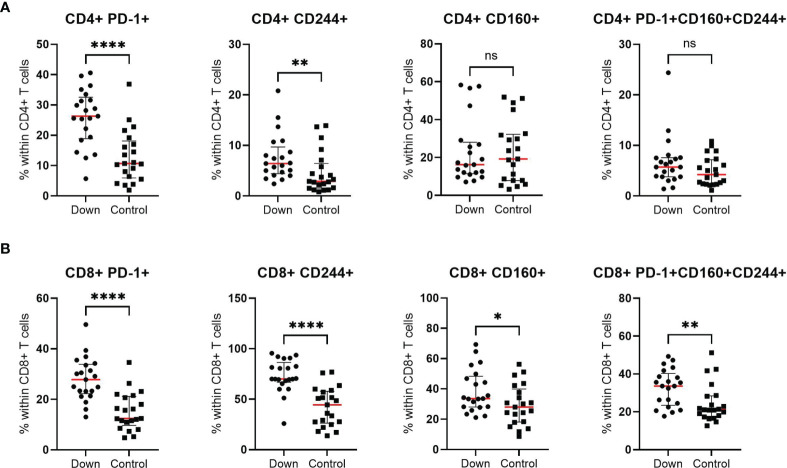
T cell exhaustion of CD4+ and CD8+ cells in 21 children with Down syndrome and 21 healthy age-matched controls. **(A)** Increased expression of PD-1+ and CD244+ and similar levels of CD160+ and PD-1+CD160+CD244+ co-expression in CD4+ T cells. **(B)** Elevated levels of all exhaustion markers (PD-1, CD160 and CD244) in CD8+ T cells. Median and interquartile ranges are indicated. *p <0.05; **p < 0.01; ****p < 0.0001; ns p > 0.05.

To study the level of T cell exhaustion, we examined the percentage of CD4+ and CD8+ T cells with the expression of PD-1, CD244 and CD160 and co-expression of these three markers in 21 children with DS and 21 controls. We found a significant higher percentage of CD8+ T cells with expression of all single markers in children with Down syndrome (**Figure 5B**). The biggest increase was seen in CD8+ T cells expressing PD-1 (mean 28.0 ± 1.9 in DS versus 15.2 ± 1.6 in controls) and CD244 (mean 73.2 ± 3.6 versus 42.4 ± 4.2). Interestingly, CD8+ T cells with co-expression of all three markers (CD8+PD-1+CD160+CD244+) were 30% higher in children with DS compared to healthy age-matched controls (mean 32.8 ± 2.1 and 24.7 ± 2.2, respectively). For CD4+ T cells, the percentages of PD-1+ and CD244+ expression were elevated in children with Down syndrome (mean 25.9 ± 2.0 and 7.6 ± 1.0, respectively), compared to controls (mean 12.9 ± 1.9 and 4.6 ± 0.9). The levels of expression of the single receptor CD160+ and co-expression of all three inhibitory receptors, CD4+PD-1+CD160+CD244+ T cells, were similar for children with Down syndrome and healthy controls (**Figure 5A**). We found higher levels of CD57 expression on both CD4+ and CD8+ T cells in DS, suggesting an increase in senescent T cells compared to healthy children (**Figure S1**). This increase was most evident in CD8+ T cells (*p*<0.0005).

## Discussion

In order to identify possible immunological alterations that could contribute to the clinical phenotype of children with Down syndrome, we explored the extent of hyperphosphorylation of the PI3K/AKT signalling pathway and T cell exhaustion and compared this with healthy age-matched individuals. We found that pAKT was increased for CD4+ T cells and B cells in children with DS. Interestingly, total AKT was increased even more than pAKT and was significantly elevated in all different subsets. In addition, increased expression of inhibitory markers PD-1, CD244 and CD160 on CD8+ T cells and increased PD-1 and CD244+ expression on CD4+ T cells suggest the presence of T cell exhaustion. We hypothesize that the combination of increased pAKT and T cell exhaustion could offer an explanation for several common clinical conditions associated with trisomy 21, including auto-immunity, increased susceptibility for infections, and haematological malignancies.

Previous studies show that children with DS have normal levels of NK-cells, but reduced total B and T cells (6). Within the memory B cells compartment, children with DS have lower natural effector and memory B cells and a different shift in the memory B subsets with increased IgM and decreased IgA and IgG memory B cells (17). For T cells, both CD4+ helper and CD8+ cytotoxic T cells are decreased in DS (18). These lymphocyte subset distributions are in line with the findings in our cohort. The immunophenotypic alterations in children with DS can be caused by disturbances in various processes, such as a change in the development of peripheral (switched) memory B cells or by an increased apoptotic state of T cells.

The combination of T cell exhaustion, immunodeficiency, auto-immunity and the predisposition to develop haematological malignancies has previously been described in APDS (8–11). APDS is characterized by a gain-of-function mutation in PI3Kδ enzyme, leading to the activation of the PI3K/AKT cascade. To further examine the role of the AKT signalling pathway in Down syndrome, we performed a phospho-flow assay in T cell subsets (CD3+, CD4+ and CD8+) and B cells (CD20+). The levels of pAKT were significantly increased in CD4+ T cells and CD20+ B cells of children with DS. To our best knowledge the PI3K/AKT/mTOR pathway dysregulation has never been examined in immune cells of individuals with DS. However, the hyperactivation of this pathway has been described in neurological cells in the frontal cortex, the hippocampus and fibroblasts of individuals with Down syndrome, and in the hippocampus of mouse models of Down syndrome (19–24). The reason for the increased levels of AKT and pAKT in Down syndrome is not clear. For APDS, the pAKT increase is caused by a gain-of-function mutation in the PI3K3CD gene, which increases p110δ activity. The δ isoform is mainly found in leukocytes. We did not examine the different isoforms, but as previous studies also found increased AKT in neural tissues (19–22), it is possible that other isoforms and therefore other tissues are also affected in DS. The AKT gene is not located on chromosome 21. However, trisomy 21 causes alterations in multiple genetic mechanisms which could also include various mediators of the PI3K/AKT/mTOR pathway. One of these regulators is PTEN, which is an inhibitor of the PI3K pathway. A study by Volk et al. showed an inhibition of the OTUD5 gene in individuals with trisomy 21, could lead to the downregulation of p53 (25). Because p53 cooperates with PTEN, its downregulation can cause lower PTEN activity and therefore lessen the inhibition of the PI3K/AKT/mTOR pathway. The role of increased PI3K/AKT activity has previously been described in patients with PTEN deficiency (26). A reduction of PTEN levels has also been found in the fetal DS brain (27). In contrast to these possible explanations for increased pAKT in DS, some *in vitro* studies show factors that might downregulate the AKT pathway. For example, increased expression of tetratricopeptide repeat domain 3 (TTC3) which facilitates the ubiquitination of pAKT (28, 29). However, our findings show a clear increase in AKT and pAKT, which indicates that the factors which positively regulate this pathway are likely more prominent in DS. We hypothesize that decreased PTEN expression might play a bigger role in the increased pAKT levels in Down syndrome. This should be examined in futures studies.

Some reports describe an increase of apoptosis for both T and B cells in DS (30, 31). Apoptosis of T cells may have been preceded by (severe) T cell exhaustion. PD-1 is an important regulator that is involved in T cell exhaustion. An elevated expression of PD-1 and other markers as CD244 and CD160 can indicate increased T cell exhaustion. Schoch et al (7) showed an increase of PD-1 expression on CD4+ and regulatory T cells in children and adolescents with Down syndrome. Another study showed increased PD-1 expression on Tfh-cells (32). In order to examine T cell exhaustion in more detail, we analysed the expression of inhibitory markers PD-1, CD244 and CD160 on CD4+ and CD8+ T cells. Similar to APDS, we found an increased expression of PD-1 and CD244 in CD4+ T cells and an increase of every single inhibitory marker and their co-expression on CD8+ cells. These findings suggest an increase in T cell exhaustion in children with DS, especially for CD8+ T cells. T-cell exhaustion and senescence appear to be related phenomena as shown by increased CD57 expression of T-cells in DS. Recently it has been shown that premature senescence in DS thymocytes plays a key role in the pathogenesis of immune defect in DS in the context of increased oxidative stress and epigenetic regulation (33). Accelerated aging in DS thymocytes has likely contributed to the T-cell exhaustion and senescence we observed in our cohort. We do not have a good explanation for our finding that AKT hyperphosphorylation was present in CD4+ T cells, rather than the exhausted CD8+ subset. This discrepancy has to be explored in future studies. Blood samples were taken at the moment that children did not experience infections, so it is unlikely that T cell exhaustion reflected a status of (recurrent) infections in DS.

Children with APDS benefit from treatment with mTOR inhibitor Sirolimus or selective PI3Kδ inhibitors (34–36). As their immunophenotype resembles those of patients with Down syndrome, we hypothesize that these therapies might also be beneficial in reducing the complications of children with DS and significant immunodeficiency or auto-immunity. However, because the pathophysiological mechanism of increased AKT seems to be different in both diseases, it is debatable to what extent these treatments could be effective. Treatment with mTOR inhibitors or PI3Kδ inhibitors for patients with Down syndrome should be explored in future research, but only if justified by the clinical severity of the associated complications.

A limitation of our study is that we examined a relatively small cohort of patients and therefore could not perform a meaningful subgroup analysis in different age groups. Because of limitations of the available blood volumes, we could not confirm AKT hyperphosphorylation by Western blot. In addition, we were not able to directly compare DS and APDS patients. However, the previous reports of AKT hyperphosphorylations in neural tissues and fibroblasts in DS and the clinical and immunological similarities with APDS are in line with our results.

In conclusion, we showed that children with Down syndrome have hyperphosphorylation of AKT and increased total AKT in B cells and CD4+ T cells. In addition, an increased expression of inhibitory receptors of CD4+ and CD8+ T cells, suggests T cell exhaustion. These alterations might contribute to the increased susceptibility to infections, auto immunity and haematological malignancies.

## Data Availability Statement

The raw data supporting the conclusions of this article will be made available by the authors, without undue reservation.

## Ethics Statement

The studies involving human participants were reviewed and approved by MREC Zuidwest Holland. Written informed consent to participate in this study was provided by the participants’ legal guardian/next of kin.

## Author Contributions

DP, GD, and MB designed the study. DP wrote the first draft of the manuscript, analysed the data and was responsible for the study logistics. IP-K and DW performed the immunological analysis. MRu, RC, MRa, FF, and GZ helped with study logistics and enrolment of subjects. MB and GD supervised the study. All authors approved the final manuscript.

## Funding

This study was supported by the Haga Teaching hospital research fund and the Elisabeth von Freyburg Foundation.

## Conflict of Interest

The authors declare that the research was conducted in the absence of any commercial or financial relationships that could be construed as a potential conflict of interest.

## Publisher’s Note

All claims expressed in this article are solely those of the authors and do not necessarily represent those of their affiliated organizations, or those of the publisher, the editors and the reviewers. Any product that may be evaluated in this article, or claim that may be made by its manufacturer, is not guaranteed or endorsed by the publisher.
