# Supplementary material for: AKT Hyperphosphorylation and T Cell Exhaustion in Down Syndrome
Source: Front Immunol. 2022 Feb 10;13:724436. doi: 10.3389/fimmu.2022.724436 (PMC8866941; doi:10.3389/fimmu.2022.724436)
Supplement: Supplementary file 1 [file DataSheet_1.docx]

Supplementary Material

# 1. Methods

*Flowcytometric immunophenotyping*

Flowcytometric immunophenotyping was performed on fresh blood using BD Canto-II FACS. Analysis was done with BD FACSDIVA software. Antibodies for the following markers were used: CD45RA BV510, TCRgd PE-Cy7, CD3 APC, IgG PE, CD24 APC-AF750, CD21 APC, CD27 BV421, CD38 FITC) (All BD Biosciences), CD4 BV510, CD28 PERCP-Cy5.5IgD PerCP-CY5.5, CD5 PE, IgM BV510) (All Biolegend, San Diego, CA, USA), CD38 APC-AF750, CD19 PC7 (All

Beckman Coulter, Brea, CA,USA), CD8 APC-AF750, IgE FITC (All Antibodychain

International B.V.), CCR7 PE, IgA PE, IgA FITC (All Miltenyi Biotech BV. Bergisch

Gladbach, Germany), CD45RO FITC (Exbio, Vestec, Czech Republic), CD8 FITC, smIgD

FITC, CD16 PE, CD56 PE, CD4 PerCP-Cy5.5, smIgM PerCP-Cy5.5, CD19 PE-Cy7, TCRgd

PE-Cy7, CD3 APC, CD45 APC-C750 (Euroflow PIDOT Tube from Cytogonos, S.L.

Salamanca, Spain).

*Exhaustion*

After thawing frozen PBMCs, samples were washed and stained with CD45RA BV510, CD279 BV711, CD160 FITC, CD197 PE-CF594, CD4 PE-Cy5.5, CD3 APC (All BD Biosciences), CD57 BV605, CD244 PE (All Biolegend, San Diego, CA, USA), CD8 APC-AF750 (Antibodychain International B.V) and CD19 PC-7 (Beckman Coulter, Brea, CA,USA). Analysis was performed using BD FACSDIVA software. To examine the extent of T cell exhaustion, the expression of inhibitory markers PD-1, CD244 and CD160 and their co-expression were examined on CD4+ and CD8+ T cells. The marker CD57 was used to identify senescent T cells or terminally differentiated T cells with reduced proliferative capacity.^1,2^

References:

1. Kared H, Martelli S, Ng TP, Pender SL, Larbi A. CD57 in human natural killer cells and T-lymphocytes. Cancer Immunol Immunother. 2016;65(4):441-452.

2. Brenchley JM, Karandikar NJ, Betts MR, et al. Expression of CD57 defines replicative senescence and antigen-induced apoptotic death of CD8+ T cells. Blood. 2003;101(7):2711-2720.

# 2. Supplementary Tables

# Supplementary Table 1 Baseline characteristics of children with Down syndrome and age-matched healthy controls.

|  | | Down syndrome  (n=22) | Controls  (n=21) |
| --- | --- | --- | --- |
| Age (y; range) | | 5; 1-11 | 5; 1-12 |
| Male | | 16 (72.7%) | 20 (95.2%) |
| Medical history^#^ | |  |  |
|  | None significant | 4 (18.2%) | 14 (66.7%) |
|  | Cardiac pathology* | 14 (63.6%) | 0 (0%) |
|  | Wheezing/asthma | 2 (9.1%) | 1 (4.8%) |
|  | Severe LRTI needing hospitalization | 8 (36.4%) | 0 (0%) |
|  | History of ENT surgery | 13 (63.6%) | 3 (14.3%) |
|  | Laryngomalacia | 2 (9.1%) | 0 (0%) |
|  | Celiac disease | 2 (9.1%) | 0 (0%) |
|  | Hypothyroidism | 1 (4,5%) | 0 (0%) |
|  | Other | 2 (9.1%) | 2 (9.5%) |
| Fully vaccinated^$^ | | 16 (88.9%) | 19 (95.0%) |
| Reason for operation | |  |  |
|  | Urological | NA | 16 (76.2%) |
|  | Surgical | NA | 5 (23.8%) |

Values are expressed as median (continues data) or count and (%) (categorical data) unless otherwise indicated. NA = not applicable. ^#^Patients can have multiple problems in their medical history. *Resolved or current cardiac comorbidity. ^$^ Percentage based on the number of subjects in whom vaccination status was known.

# Supplementary Table 2, True counts of lymphocyte subsets in Down syndrome and age matched healthy controls

Down syndrome

Age in years leukocytes* lymphocytes* T-cells* B-cells* NK-cells*

1 4,30 2,07 1,67 0,41 0,13

1 6,20 1,88 0,97 0,26 0,25

3 2,90 1,22 0,49 0,34 0,08

3 6,00 3,35 1,90 0,58 0,07

3 8,00 1,90 1,26 0,29 0,29

3 4,80 1,63 1,27 0,16 0,14

3 7,70 6,12 2,43 0,40 0,10

3 3,50 2,19 1,78 0,12 0,30

4 4,30 2,99 1,69 0,10 0,26

5 6,90 3,58 2,68 0,52 0,31

5 4,30 2,97 1,99 0,14 0,21

6 5,10 4,11 1,38 0,36 0,26

6 9,10 4,30 1,34 0,20 0,18

6 5,10 1,69 1,60 0,21 0,15

6 7,30 2,59 1,89 0,71 0,37

6 5,70 0,84 1,03 0,18 0,07

8 5,30 2,28 1,46 0,20 0,44

8 8,50 2,71 3,03 0,23 0,31

9 4,80 2,11 0,17 0,02 0,02

10 4,90 3,85 1,55 0,34 0,54

12 4,50 1,86 1,76 0,21 0,21

12 4,60 1,48 1,37 0,07 0,19

Healthy controls

Age in years leukocytes* lymphocytes* T-cells* B-cells* NK-cells*

1 11,30 7,06 6,36 1,65 0,62

1 10,30 5,84 1,82 1,20 0,26

2 7,90 5,34 2,82 0,72 0,38

3 7,70 3,14 2,42 1,10 0,18

3 8,30 3,22 2,26 0,52 0,13

3 4,60 2,77 1,04 0,30 0,09

3 6,90 2,75 2,13 0,42 0,15

4 4,90 2,64 1,95 0,28 0,40

4 ND ND ND ND ND

6 6,40 3,63 2,25 0,41 0,18

6 4,40 2,57 1,40 0,46 0,09

6 4,10 1,90 2,00 0,45 0,11

6 5,40 3,93 1,72 0,39 0,16

7 6,50 2,02 1,27 0,19 0,02

7 6,60 2,79 1,59 0,04 0,67

7 7,30 2,48 1,59 0,42 0,31

10 2,80 1,46 1,16 0,28 0,43

11 4,80 1,46 1,26 0,25 0,88

11 5,80 3,22 2,60 0,57 0,27

11 5,20 2,66 1,76 0,42 0,36

12 5,40 3,96 1,68 0,29 0,54

, *x10^9^/L. ND; no data: flowcytometric immunophenotyping data of one healthy subject was
not available because of a too small extracted blood volume.

# 3. Supplementary Figures


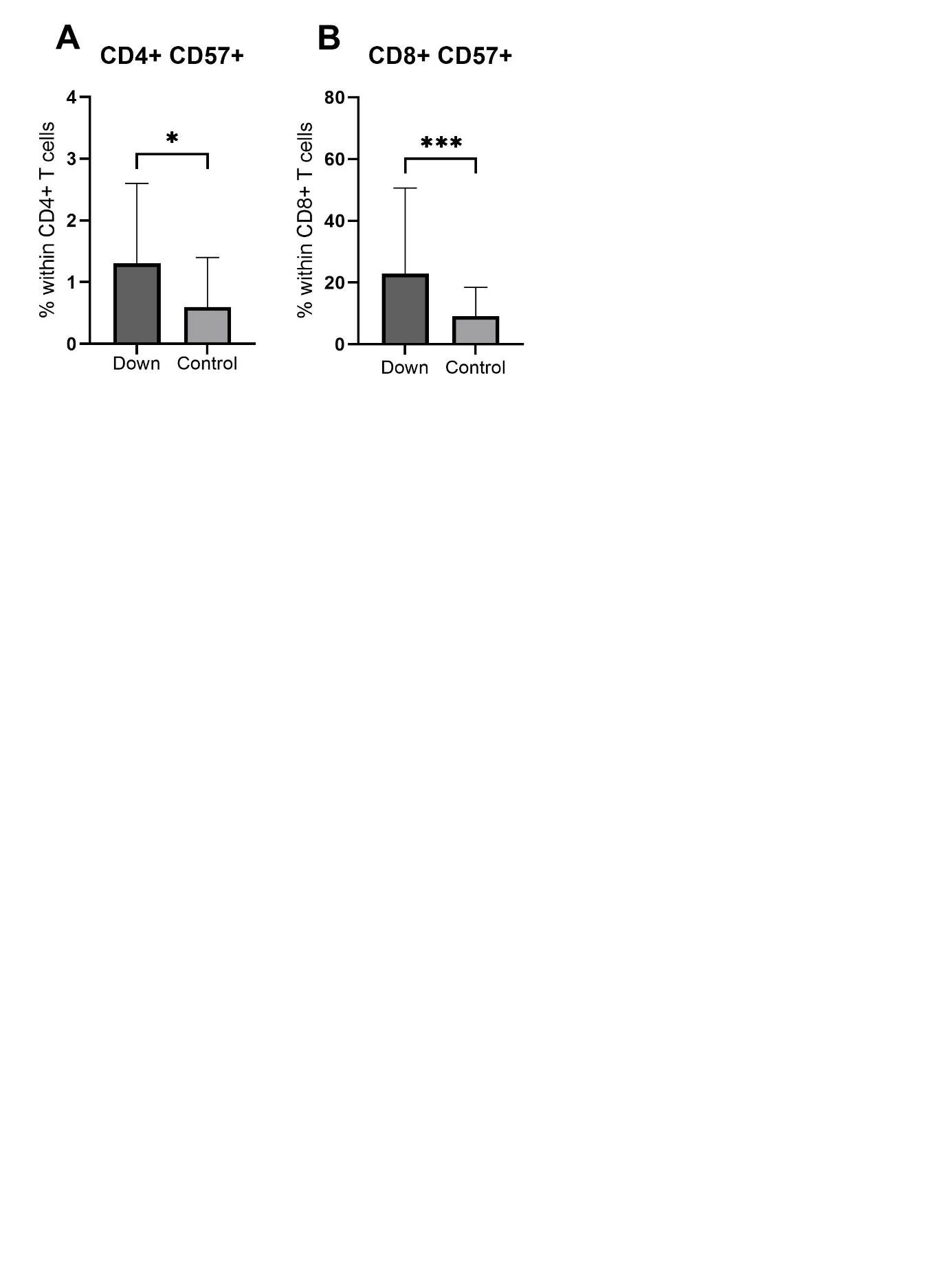


**Supplementary figure 1 – Expression of senescence marker CD57 on CD4+ and CD8+ T cells in children with Down syndrome (n=21) and age-matched healthy controls (n=21).** A. Increased expression of CD57 on **A** CD4+ T cells and **B** CD8+ T cells in children with Down syndrome (*p*=0.0117 and *p*=0.0004). Median and interquartile ranges are indicated. * *p*<0.05; ** *p*<0.01; *** *p*<0.001; **** *p*<0.0001; *ns* *p*>0.05

**
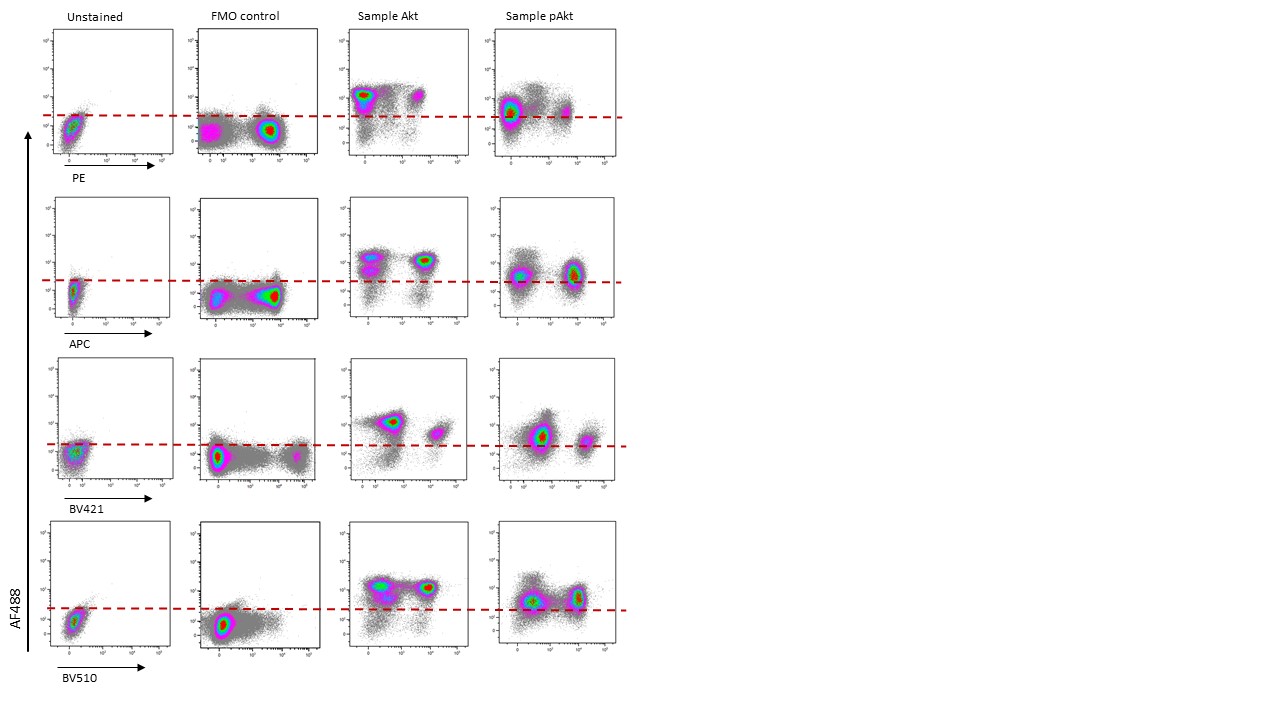
**

**Supplementary figure 2**. **FMO controls to check for background staining**. For every fluorochrome used for definition of a population (PE, APC, BV421 and BV510) for Akt and pAkt staining a separate FMO control was made. A representative control is displayed.
